# Vaccination Is Not Enough: Understanding the Increase in Cases of COVID-19 in Chile despite a High Vaccination Rate

**DOI:** 10.3390/epidemiologia2030028

**Published:** 2021-08-24

**Authors:** Sabrina Lanzavecchia, Katharina Johanna Beyer, Sophie Evina Bolo

**Affiliations:** 1 Global Studies Institute, Université de Genève, 1211 Genève, Switzerland; sabrina.lanzavecchia@etu.unige.ch (S.L.); sophie.evina@etu.unige.ch (S.E.B.); 2 Hôpitaux Universitaires de Genève HUG, Service de Médecine de Premier Recours SMPR, Unité des Urgences Ambulatoires UUA, Rue Gabrielle-Perret-Gentil 4, 1205 Genève, Switzerland

**Keywords:** Chile, COVID, coronavirus, vaccination, vaccines

## Abstract

Chile, an OECD country in the southern hemisphere, surprised the world with a high speed COVID-19 vaccination rate at the beginning of 2021. Despite this, cases reached a record high again in April 2021, and the country went back to a state of emergency. The reasons for this are multiple, complex, and interconnected. A feeling of false safety with the beginning of vaccination, the appearance of new more transmissible variants, too early relaxation of non-pharmacological measures at a point of vaccination below herd immunity, and vaccination in a high prevalence setting, appear to be main reasons for the resurgence. However, the political context and the socio-economic inequalities in Chile also play an important role, and are more difficult to measure and to compare with other countries. In conclusion, the Chilean example is a warning sign not to count on vaccination figures alone, and to maintain some of the previous non-pharmaceutical strategies to contain the pandemic.

## 1. Introduction

Chile is a democratic presidential republic situated along South America’s western coast with a total population of around 19 million, and is a high-income country, member of the Organisation of Economic Cooperation and Development (OECD). However, income and other inequalities are higher than in any other OECD country—65% higher than the OECD average [1]. Since October 2019, socio-economic inequalities have triggered a wide movement of protests, demanding a change in the country’s political and social direction, and denouncing the high costs of healthcare and poor funding of education [2].

Chile’s health system consists of a dual sub-system of public and private health insurance and health care service provision. The public system focuses on primary health care, deeply connected with local communities, while the private system is specialized in secondary and tertiary services. This mixed health system contributes to inequalities in access to healthcare facilities, preventive care, and treatment [3].

The first case of the SARS-CoV-2 virus in Chile was confirmed by the Minister of Health on 3 March 2020 [4]. The World Health Organisation (WHO) declared South America as the new epicentre of COVID-19 on 22 May 2020 [5]. By reaching 6727 confirmed daily new cases and above 200 confirmed daily deaths by mid-June, Chile became the sixth most affected country in the world. Over the following months, the number of daily new cases and deaths began to decline slowly, stabilizing at under 1400 daily cases and 50 daily deaths until 2 December 2020 [6]. 

In April 2021, Chile experienced again a major epidemic with over 6000 new confirmed cases per day and a record high of 7320 confirmed cases reported on 14 April 2021, reaching 1.20 million cumulative cases and 26,353 cumulative confirmed deaths as of the end of April [6]. This was despite the fact that 35.2% of the population had been fully vaccinated with two doses. Only Israel and the United Arab Emirates had fully vaccinated more of their population, with 58.9% and 38.8%, respectively [6]. 

The purpose of this article is to present and analyse available data and identify what factors may be associated with this high transmission rate and what lessons can be learnt from the Chilean response to the COVID-19 pandemic. First, we will give a general overview of the demographic, political, economic, geographic, and climatic characteristics of the country. We will then describe the evolution and compliance of non-pharmaceutical measures undertaken by health authorities since the first recorded case in Chile on 3 March 2020; Chile’s strategic and unique vaccine procurement policy, and the implementation of Chiles’ national vaccination plan and the social and economic impact of these measures. Finally, we will try to understand why vaccination alone in Chile is not enough to prevent rising infection rates and to end the pandemic.

## 2. Methods and Case Presentation

### 2.1. Methods

The three authors independently conducted an online literature search between 15 March 2021 to 30 April 2021 in English and Spanish. We searched for our keywords “Chile; COVID; coronavirus; vaccination; vaccines” in Google Scholar and Pubmed, regularly checked and collected data from the coronavirus section of the Oxford University database “Our World in Data” related to Chile and periodically checked the website of the WHO, the Chilean government and the Ministry of Health for reports and publications related to our keywords. Finally, we followed major international and regional news websites (New York Times, BBC, Latercera, Vox) for the same keywords. The articles identified and selected by each researcher were made available to each other on a shared Dropbox folder. Regular zoom meetings took place to discuss the relevance of the articles and checked for the verifiability of the data. Criteria for inclusion were in the first line the focus on Chile. We then complemented our findings using “snowballing”, i.e., finding the original studies or governmental decisions the newspaper articles were referring to, going backwards to the beginning of the pandemic in Chile. Last, we eliminated duplicates, keeping the most original source, i.e., the scientific articles, then the governmental or WHO official reports, and lastly the press articles. To create the graphs, we used the Creative Commons—By Attribution (CC-BY) licensed open source Our World in Data. 

### 2.2. Case Presentation

#### 2.2.1. Demographic, Political, and Economic Characteristics of the Country

Chile is situated along South America’s western coast. It extends approximately 4300 km (2700 miles) north to south and encompasses nearly all climate zones [7]. The total population of the country is around 19 million, which translates into a density of only 25.7 people per sq. km. In total, 87.6% of the population lives in urban areas, of which 35% in the capital city Santiago [8]. In terms of age-group distribution, the population aged 60 and over was 17.4% in 2020 up from 13.3% in 2010, due to both improved health care conditions and lifestyle predominantly in the urban population [7], while the percentage of the population under 15 was 19.2% in 2020, down from 22.1% in 2010, reflecting a decline in fertility rates [8,9].

Chile is a democratic presidential republic. Local government is divided in 15 administrative regions, including the metropolitan region of Santiago. Since 2019, Chile has experienced social conflict with widespread discontent and demand for reforms. This led the government to order a state of emergency and implement a mandatory curfew [10]. On 25 October 2020, Chileans voted in favour of a new constitution to rewrite the one drafted under General Augusto Pinochet in 1973. The vote to select 155 Chileans who will form the convention and draft the new constitution was scheduled for 11 April 2021 but had to be postponed to 15–16 May 2021, due to the deterioration of the COVID-19 pandemic.

The Chilean economy is based on agriculture, fishing, forestry, and mining resources [7]. Chile is the world’s largest producer and exporter of copper. Thanks to many bilateral and regional trade agreements the country entered into in the last twenty years, Chile has become one of the most successful economies in South America [7]. However, income and wealth inequalities are very high, with one percent of the population controlling 26.5% of the country’s wealth, while 50% of low-income households access only 2.1% [1]. This makes Chile one of the three most unequal countries in Latin America. In 2020, gross domestic product (GDP) per capita was USD 15900 and as of 2019, the GINI coefficient on the degree of inequality in wealth distribution was 44.4. [8,11]. 

#### 2.2.2. Healthcare System

The Chilean health system has undergone improvements in recent decades and is financed both by public funds covering the entire population, and by private funds, including out-of-pocket expenditures and premiums paid for private health schemes [3]. 

The National Health Service is responsible for the provision of primary care services by local health facilities run by municipalities. The public system’s focus on primary care has allowed to respond to basic needs of the population and to improve health outcomes. The private sector, which serves a smaller portion of the population, mostly focuses on secondary and tertiary care through a network of outpatient facilities, providing higher-quality care and utilising more advanced technology, comparable to the standards of other developed countries [3].

Various public health programs provide vaccinations and complimentary nutrition, maternal, and childcare, and address chronic illnesses. Nevertheless, despite significant improvements across several health indicators in the past decades, Chile’s health system still faces some major challenges to deal with the needs of an ageing population involving an increase in chronic non-communicable diseases coupled with a lack of specialised health professionals and equipment, especially in the public sector, which serves about 78% of the population [3].

Access to care is especially inadequate among those living in poverty, indigenous populations and in rural areas, with barriers related to living far from health services and unaffordable transportation costs. Additionally, health care providers concentrate in urban areas, resulting in inequitable geographic distribution of physicians [12]. 

## 3. Results

### 3.1. Management and Outcome of the COVID-19 Outbreak

On 18 March 2020, the Ministry of Health (“MINSAL” for its Spanish acronym) issued a nationwide health alert (“Health Alert”), granting the Chilean health authorities a series of extraordinary powers for one year in order to confront and prevent the spread of the novel coronavirus and to centralise the measures implemented during the crisis. Maximum prices for certain pharmaceutical or health products, medical services, and lease of property were set. The same day, President Piñera declared a constitutional state of emergency for 90 days. This was extended later for another 90 days. It allowed the government to restrict or even suspend individual and social rights in order to restore normalcy in the shortest possible time [10].

No national lockdown was established in Chile, unlike in neighbouring Argentina and Peru, but rather localised lockdowns in different cities and neighbourhoods. A night curfew (22 p.m.–5 a.m.) was implemented throughout the country. Most schools were closed from March until January 2021. Non-essential shops, bars, and restaurants closed, but reopened at the end of 2020 to boost the economy during holidays. As in most countries, cultural, sport, and public events were cancelled.

Chile started public testing available to anyone, including asymptomatic people, by mid-November 2020. It has since then been the country with the widest and most consistent testing strategy in the region. The testing rate per day increased from around 1.8 tests per thousand people until November 2020, to around two at the beginning of 2021, up to 3.5 at the end of 30 March [6]. The first increase can be attributed to the implementation of the broader testing strategy, whereas the later increase might be linked to the spike in prevalence with a higher positivity rate in a setting of comprehensive tracing. As per 30 April 2021, there were 683 tests performed per 1000 people, which is in the range of most of the European countries [6]. Quarantines were imposed on those who were tested for COVID-19 until they were notified with the results, as well as for those in close contact with a person diagnosed with COVID-19. A 14-day isolation was mandatory for people diagnosed with COVID-19 [13]. People entering the country, regardless of their country of origin, had to comply with quarantine measures for 14 days. Failure to comply with measures imposed by the authority was monitored and punished according to the provisions of the Criminal Code, when applicable [14]. 

Borders were closed from 16 March 2020 and reopened on 7 January 2021, but closed again on 31 March 2021, following the second declaration of state of emergency [15].

In addition, state funds were allocated for non-pharmaceutical intervention measures as follows: Chilean pesos (CLP) 2.3 billion (around USD 3.3 million) in COVID-19 research funds, CLP 72 million (USD 100,000) in competitive funds for mental health development, CLP 800 million (USD 1.1 million) for the innovation and entrepreneurship challenge to create personal protection equipment (PPE), CLP 1.5 billion (USD 2.1 million) to support the PCR diagnostic lab network, and CLP 500 million (USD 700,000) from the Chilean Economic Development Agency (Corfo) to promote the production and scale-up of mechanical ventilators [16].

Presence on social media, with campaigns on self-hygiene measures (hands washing, self-quarantine, etc.) including a comprehensive site for vaccination (#Yomevacuno), was also an important part of the strategy.

### 3.2. Economic and Social Impact

The pandemic impacted Chile at a particularly complex social and political time. The 2019–2020 protests exposed the vulnerability of the socio-economic system, with protesters demanding a change in the country’s political and social direction. In this context, the COVID-19 pandemic plunged the economy into the worst recession in decades. GDP contracted 6.0% in 2020, although a loosening of lockdown measures allowed a partial recovery towards the end of the year 2020. Over one million jobs were lost, affecting mostly women and workers in commerce, agriculture, and hospitality, further undermining the fragile middle class. Public debt rose from 28% in 2019 to 33% in 2020. Poverty is expected to have increased from 8.1% to 12.2%, with about 780,000 people expected to have fallen into poverty despite one of the largest policy responses in the region, including cash transfers, a job retention scheme, tax deferrals and reductions, liquidity provisions and guarantees, and early withdrawals from pension funds [17].

The establishment of a constitutional state of emergency for public health purposes only a year after similar measures had been taken for social unrest further deepened the pre-existing inequalities. The most vulnerable, i.e., those experiencing poverty, overcrowded living conditions and malnutrition and relying on informal sector jobs were the hardest hit by the pandemic, a reality that reflected the wider debate in Chile about the country’s inequalities [18,19]. Despite Chile having relatively low levels of informal labour (30%) compared to Latin America (53%), many women and men could not stay at home since they need to earn an income to feed themselves and their families, as most of them cannot rely on income replacement or savings [20]. The pandemic, in general, has also eclipsed regular health care services, and now the vaccination program might continue to do so, especially when it involves child and maternal health. The primary health care strategy is facilitating the rollout of the vaccines at the expense of essential services [21].

### 3.3. Epidemiological Situation

**Figure 1:**
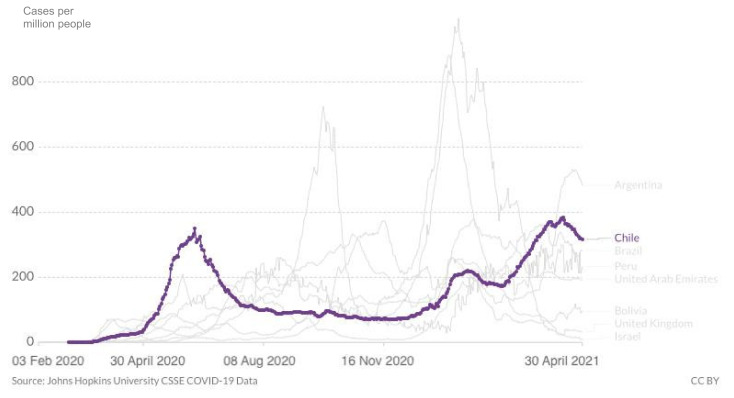
Number of daily new confirmed COVID-19 cases per million people in Chile from the first notified case until 30 April 2021, including comparison with the region (Argentina, Brazil, Bolivia, Peru) and other leading vaccination nations (Israel, UAE, UK). www.ourworldindata.org (accessed on 1 May 2021).

**Figure 2:**
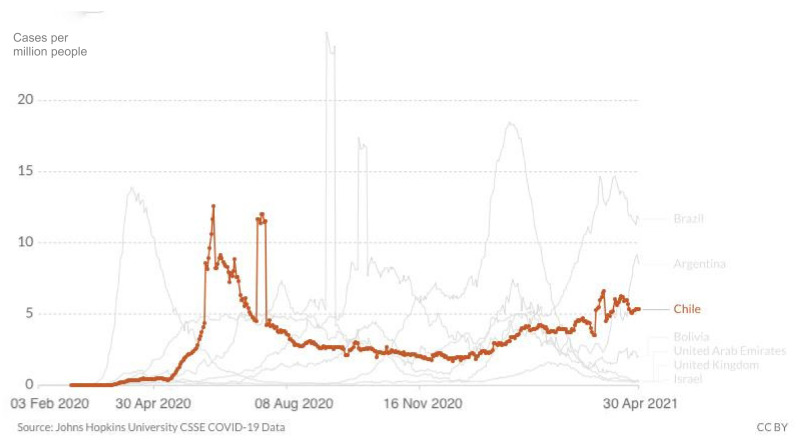
Number of daily new confirmed COVID-19 deaths per million people in Chile until 30 April 2021, including comparison with the region (Argentina, Brazil, Bolivia, Peru) and other leading vaccination nations (Israel, UAE, UK) www.ourworldindata.org (accessed on 1 May 2021).

**Figure 3:**
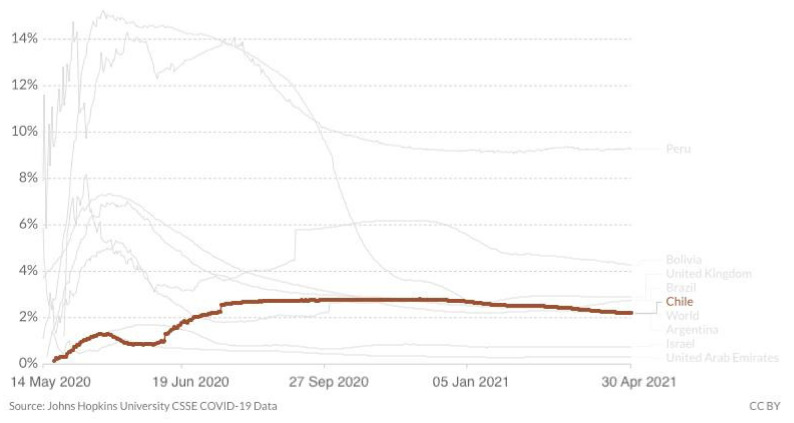
Case fatality rate of COVID-19 in Chile until 30 April 2021, including comparison with the region (Argentina, Brazil, Bolivia, Peru) and other leading vaccination nations (Israel, UAE, UK) www.ourworldindata.org (accessed on 1 May 2021).

At the end of April, cases of SARS-CoV-2 were still surging in Chile despite the country having one of the most successful vaccine purchasing strategies and rollouts in the world. As of 30 April 2021, the country experienced a major epidemic with 1.20 million cumulative cases, 26,353 cumulative confirmed deaths and 6000 new confirmed cases per day, down slightly from the record high of 7320 confirmed cases reported on 14 April 2021 (Figure 1). The death rate also rose and was slightly above 5 daily deaths per million people, up from less than 2 at the beginning of 2021. Still, they never came close to the numbers in June 2020, above 10 (Figure 2). Regarding the case fatality rate, the trend is slowly declining since January 2020 (Figure 3), which corresponds to the beginning of vaccination. When broken down to age groups, the decline is attributed to the older age groups, whereas April 2020 saw a rise in the fatality rate for young adults.

### 3.4. National Vaccination Plan

Chile was able to implement a rapid and organised immunisation campaign relying on an existing strong health care infrastructure, a century-long tradition of mass immunisation campaigns, also in the context of epidemics arising from severe natural disasters, i.e., earthquakes and tsunamis that periodically have hit the country, and on the use of electronic records [21]. 

#### 3.4.1. Vaccines Purchasing Strategy

The different ministries of Sebastian Piñera’s government—including science and technology, health, and international affairs—all coordinated on a plan to seek out safe and effective vaccines. The aim was to achieve a highly diverse portfolio of vaccines, relying on different technologies at different stages of development to hedge risks and to overcome the existing situation of market distortion and limited supply. Unlike other countries or initiatives that decided to rely only on one or few vaccines manufactured by American or European producers, Chile strategically decided not to align itself geopolitically and, instead, negotiated contracts with as many vaccine producers as possible [21].

Some experts also credited Chile’s openness and free trade policies under the administration of the centre-right president Sebastián Piñera for the country’s success in negotiating and signing letters of intention and pre-purchase contracts at favourable terms. He managed to purchase the Pfizer vaccine for USD 14 per dose instead of USD 19, convincing Pfizer of the country’s deteriorated economic situation due to the COVID-19 outbreak [21].

Chile also succeeded in having priority and preferential access to doses once approved by the national regulatory body, thanks to its participation in Phase III trials with different vaccine producers, initially from China. As Chilean universities already had some long standing partnerships with Chinese researchers on other vaccines [21], this also helped their access to vaccines. 

Public and private investments allowed the development of Phase III trials in Chile headed by Universidad Católica and Sinovac, involving 3000 volunteers between the ages of 18 and 65 [16]. The Government contributed CLP 2.6 billion (around USD 3.4 million) which were supplemented by CLP 1.6 billion (USD 2 million) allocated by the Confederation of Production and Commerce (CPC), and CLP 1 billion (USD 1.3 million) by Universidad Católica [16]. Universidad Católica signed an agreement with the Minister of Health to fully transfer its rights to use the vaccine once Phase III trials were successful and the use of the vaccine was approved by the national regulatory agencies [22]. 

Chile also signed Phase III trials with the Chinese manufacturer CanSino, the American Johnson & Johnson. In addition, Chile participated in the Self-financing Participant (SFP) Facility of COVAX, the global initiative co-led by the Coalition for Epidemic Preparedness and Innovations (CEPI), Gavi, and the WHO working with governments and manufacturers, to ensure that COVID-19 vaccines become available worldwide to both high-income and lower-income countries.

#### 3.4.2. Characteristics of Available Vaccines

As of the end of April 2021, four different vaccines received emergency approval by the Instituto de Salud Pública (Public Health Institute) in Chile: Pfizer/BioNTech, Sinovac, Oxford AstraZeneca, and CanSino. Janssen Pharmaceuticals (Johnson & Johnson) has carried out Phase III trials in Chile but its vaccine had not received the authorisation for emergency use by the end of April. Phase III trials for Sputnik V vaccine by Gamaleya Research Institute have started in Chile (Table 1).

Chile has signed pre-purchase agreements for 90.2 Mio vaccine doses from vaccine manufacturers that have conducted Phase III trials in Chile or have received an authorisation for emergency use (Table 2). Chile will also receive 7.8 Mio of doses under Covax SPF Facility through the Pan American Health Organisation (PAHO) Revolving Fund for Access to Vaccines [23]. Chile has not signed a large contract with Pfizer because of the country’s lack of proper infrastructures and logistics to deliver and store their vaccines at the required temperature.

#### 3.4.3. Prioritisation of Target Groups

Chile was the first country in Latin America to start vaccinations against COVID-19 on 24 December 2020 when the first 10,000 doses of the Pfizer/BioNTech vaccine arrived. Health workers were the first to be vaccinated [24]. The mass vaccination campaign started the first week of February with Sinovac vaccines and targeting elderly (age 90 above and descending from there), risk groups categories (age 46–59), teachers and educators (above age 26). After the first trimester vaccination was open to everyone above age 46 [25].

Government authorities changed the original vaccination plan set out by the Minister of Health and announced that foreigners not legally resident in the country were not eligible to be vaccinated, since they wanted to avoid the so-called “vaccination tourism” as happened in Dubai [24]. Foreigners with a tourist visa, as well as illegal immigrants or workers had no right to get vaccinated. Only Chilean nationals, foreign nationals with permanent or temporary residency in the country were allowed to have access to vaccination [24]. This measure directly concerned thousands of illegal immigrants that came into Chile from the northern border with Bolivia and was considered highly discriminatory towards vulnerable population groups by Amnesty International, as well as against recommendations to stop the COVID-19 outbreak [26].

#### 3.4.4. National Vaccination Campaign

There are three important factors for a successful vaccination campaign, according to Luis F. López-Calva, the regional director of the UN Development Program: first, to have the financial means to acquire vaccines; second, a good strategy for distribution; and third, to have the institutional capacity and the governmental structure to implement it. In his opinion Chile fulfils these criteria [27].

Chile has a history of successful vaccination campaigns, relying on a robust primary health care system and electronic records that keep track of when people receive their shots and when they are due for their second dose, no matter where they are. The #YoMeVacuno (“I get vaccinated”) cards that people have been posing with on social media are another advantage [21]. All people have to do is find the most convenient vaccination site in their region or community and go from there. Nobody has to make an appointment; it is free and voluntary. It is the exact opposite of the residency requirement for the appointment systems, such as in the United States [21].

In order to increase the vaccination rate, 1400 new vaccination centres were put in place, using stadia, sport centres, and schools. The healthcare workforce was reinforced by employing medical students, dentists and retired doctors. The collaboration between different levels of government, as well as between the central, regional, and local government entities was essential to set up this massive organisation [24]. If Chile had a more decentralized administrative structure, such as in Brazil or Mexico, this organisation and coordination might have required much more time, which countries do not have to fight the pandemic [24]. The government’s target is to have vaccinated 80% of the adult population by June 2021 with at least one dose to 15 million people.

#### 3.4.5. Vaccination Outcome

**Figure 4:**
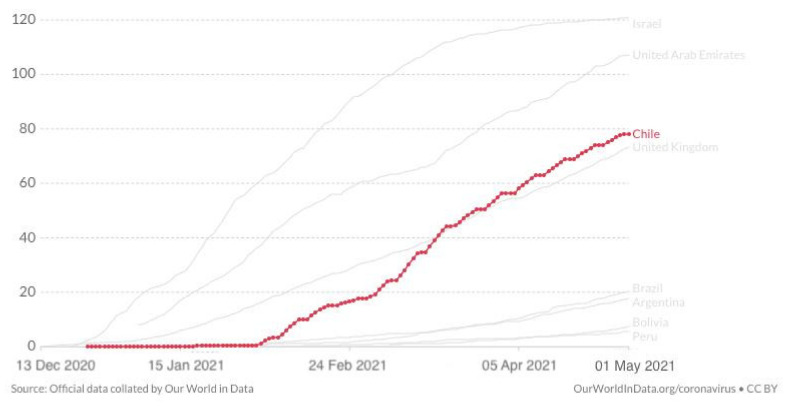
COVID-19 vaccine doses administered per 100 people until 30 April 2021, including comparison with the region (Argentina, Brazil, Bolivia, Peru) and other leading vaccination nations (Israel, UAE, UK) www.ourworldindata.org (accessed on 1 May 2021).

**Figure 5:**
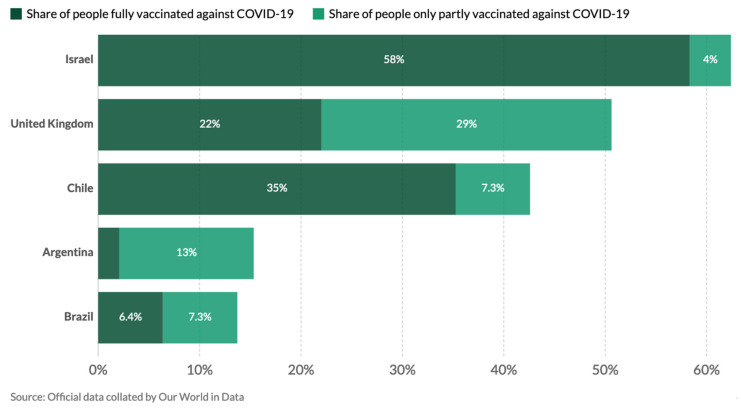
Share of the total population vaccinated against COVID-19, i.e., fully with two doses or partly with one dose, as of 30 April 2021, for Chile. Comparison with other countries in the region (Argentina and Brazil) and the vaccination leaders Israel and UK www.ourworldindata.org (accessed on 1 May 2021).

Chile started vaccinating at the end of December 2020 with an impressive speed from February onwards. As of 30 April 2021, with a target of 15,200,840 people to be vaccinated by June, 15,024,949 doses of vaccine have been administered, which means 78.13 doses per 100 people in Chile vs below 20 doses in the region (Figure 4). 8,213,066 people have received the 1st dose and 6,811,883 people have received both doses. Full vaccination coverage (person with 1st and 2nd dose) is 35.2% (Figure 5) of which 12,790,551 doses are from Sinovac, 2,190,388 doses from Pfizer, and 44,010 doses from AstraZeneca.

In addition to working to maintain its vaccination system, Chile has now decided to pursue approaches that limit the transmission of COVID-19 in the light of the resurgence of cases. The vaccination campaign is ongoing and also provides an opportunity to disseminate ongoing awareness messages that encourage behaviour that reduces the risk of transmission of the COVID-19 virus, identify the signs and symptoms of COVID-19, and indicate what to do if symptoms occur [22].

## 4. Discussion

The situation in Chile can be seen as a warning sign to the world, and we hope that lessons can be learned and applied in other contexts. This case suggests that being a vaccine champion alone is not enough to contain the pandemic. In the context of a pandemic due to a relatively new pathogen, much remains unknown about how vaccination will change the situation. Although the rapid development and deployment of the vaccine appear to be showing promising results, it is too early to draw conclusions. The reasons for the spike in infection cases despite the impressive vaccination rates are diverse, complex, and cumulative. Some of them can be found as early as the beginning of the pandemic [28,29]. The list of reasons identified in our study may, therefore, not be exhaustive.

The relaxation of travel restrictions for the Chilean summer holidays before the start of vaccination played an important role in the April resurgence. Millions of Chileans moved around the country, leading to new localized breakouts, which overwhelmed the capacity of local health services [30]. 

Misleading communication might partly explain people’s behaviour. It was probably not emphasized enough that vaccination was—at least at this stage—only another pillar in the fight against the pandemic, and primarily proven to reduce disease severity and death rate, but not transmission. As a result, many Chileans relaxed too early and abandoned other barrier measures.

Chile opened its borders on 7 January 2021 only two weeks after the start of the vaccination campaign on 24 December 2020. All neighbouring countries had a much lower vaccination rate, combined with a high prevalence. 

The emergence of new variants, especially the P1, the so-called Brazilian variant, first discovered in Manaus in November 2020 [31], seems to play an important role in Chile’s surge in new cases, according to S.M. Bueno from the Pontificia Universidad Católica de Chile, and author of the CoronaVac study [32]. P1 is estimated to be 2.5 times more transmissible than the wild variant [33,34]. 

The number of Chileans who received two doses of vaccine is an important point, especially in the context of the vaccine chosen. More than 90% of Chileans have been vaccinated with CoronaVac, which in the South American context showed an efficacy of 50–56.5% after the second dose, but only an insignificant 3% (35% CI 6.6–60.5 in the Brazilian Manaus study) after the first dose [30,32,35]. We also believe that, even in the ideal case of a vaccine providing 90% protection against transmission of the virus, the outcome is likely to be the same, since, after vaccination, people may be less cautious and meet more people than before [36].

Vaccination does not immediately slow down the transmission of infection when the virus circulation is high, according to Denise Garrett, epidemiologist at the Sabin Vaccine institute in Washington D.C. [37]. In comparison with many other countries, which saw a sharp decrease in new cases after the first and second wave, South America remained with what is considered a high epidemic region. Chile, in fact, never had an effective pandemic control and the transmission rate has always remained high [29]. In addition, vaccination of the older and more vulnerable population shifted the infection to the younger healthier population, with potentially a different and riskier behaviour, since such groups may feel less vulnerable. This can lead to higher transmission rate [38].

The vaccine rate is still far below the herd immunity threshold, which is assumed to be around 70%. To give an idea of the scale, the vaccination of all people over 18 years—the age limit for vaccine approval in Chile and most of the world by the end of April 2020—would mean a coverage of 76% of the population. 

Vaccine hesitancy can also be a concern, but it does not seem relevant in Chile, which has a more than 100-year-old history of successful vaccination campaigns, in a context of epidemics arising from natural disasters the country has been exposed to [3]. According to a recent survey, more than 72% of Chileans said they would get the COVID-19 vaccine. Even if they had preferred a US or EU vaccine, they would accept a Chinese one [21]. 

Finally, as in many other countries, after one year, there is a fatigue and, therefore, a diminishing respect of the measures implemented to contain the pandemic, such as physical distancing, mask wearing, hand washing, quarantine, isolation, limitation of movement, etc. This is, in part, especially true for the poorest and most vulnerable part of the population, who are more likely to go out to earn their living. With 30% of its workforce being in the informal sector, this is a highly relevant factor in Chile [39].

Nevertheless, vaccination seems to have a positive impact on limiting the death toll. Even though deaths occur after the peak, the figures until the end of April do not show the spike from June 2020, and the relative reduction in excess mortality in the elderly, e.g., the vaccinated, seems to support this [6]. It is worth reminding that initially the reduction in the mortality rate and severity score was the primary end point for vaccine authorisation. 

### Limitations

The limitations of this case study highlight a major issue in pandemics: the process is ongoing and highly dynamic. A screenshot can never explain the whole picture, and some unexpected lessons will continue to appear. Many variables determine the evolution, including some apparently not related to, as for example the political context and socio-economic factors, more difficult to measure.

Data available in real time does not allow to take into account all facets of the pandemic. Data on the trend of hospitalisation and ventilated patients for this period could not be found. Furthermore, study data publicly available regarding vaccines are heterogeneous, especially for the Chinese ones. This makes it difficult to estimate the part that can be incriminated to vaccines alone. 

## 5. Conclusions

South America was declared as the new epicentre of the novel SARS-CoV-2 on 22 May 2020 and by mid-June 2020 Chile became the sixth most affected country in the world, by reaching the highest number of confirmed daily new cases and of confirmed daily deaths, with 6.727 and 12.60 per million people, respectively. Despite the country having one of the most successful vaccine purchasing strategies and rollouts in the world [40], cases of SARS-CoV-2 are still surging in Chile as of 30 April 2021.

Our analysis has shown that no single intervention can be considered 100% effective, and only the combination of several interventions can sufficiently protect from catching and spreading a highly contagious disease. In the context of a new emerging disease, with a high degree of uncertainty, it seems illusory to rely on vaccines alone.

The role of leadership in such a situation is to make sure several layers of protection stay in place, and to adapt the regulations according to it. Continuing mask wearing, social distancing and travel restrictions remain important, as well as ensuring psychological and social balance. Policy makers need to take decisions on COVID-19 control strategies, considering their epidemiological outcome, as well as the associated social, and economic effects [39]. Each intervention should be adapted to the local and regional context.

## Figures and Tables

**Table 1:** Characteristics of type of vaccines available and approved for emergency use or on Phase III trials in Chile, as of 30 April 2021.

Company	Name	Country	Type	Doses	Conservation Temperature	Emergency Authorisation
Pfizer Inc-BioNTech	BNT162b2	USA/Germany	mRNA	two	−30 °C	Approved on 16 December 2020
Sinovac	Corona Vac	China	Inactivated virus	two	2–8 °C	Approved on 20 January 2021
Oxford AstraZeneca	Vaxzevria	UK/Sweden	Adenovirus viral vector	two	2–8 °C	Approved on 27 January 2021
CanSino Biologics	Ad5-nCoV	China	Adenovirus viral vector	one	2–8 °C	Approved on 7 April 2021
Janssen Pharmaceuticals (J&J)	JNJ-78436735	USA	Adenovirus viral vector	one	2–8 °C	Not approved
Gamaleya Research Institute	Sputnik V	Russia	Adenovirus viral vector	two	2–8 °C	in Phase III trials

**Table 2:** Purchased quantities, supply volumes, and delivery status of vaccine doses as of 30 April 2021.

Company	Purchased Doses In Millions	Delivered Doses In Millions (% of Purchased)	Administered Doses (% of Delivered)
Pfizer Inc-BioNTech	10	5 (50)	2.190.388 (44)
Sinovac	60	14.2 (24)	12.790.551 (90)
Oxford AstraZeneca (Covax Facility)	14.4	0.158 (1.1)	44.010 (28)
CanSino Biologics	1.8	None	None
Janssen Pharmaceuticals (J&J)	4 Mio	None	None

Data: Vaccine Procurement Database, Global Health Innovation Center, Duke University. Available online: https://launchandscalefaster.org/COVID-19/vaccineprocurement (accessed on 1 May 2021) and Pan American Health Organisation. Available online: https://www.paho.org/en/covax-americas (accessed on 1 May 2021).

## Data Availability

www.ourworldindata.org (accessed on 1 May 2021) open source under Creative Commons-By Attribution (CC-BY) license, Duke University and Pan American Health Organisation (PAHO) (accessed on 1 May 2021). No new data were created or analyzed in this study. Data sharing is not applicable to this article.
